# Pain Control Following Impacted Third Molar Surgery with Bupivacaine Irrigation of Tooth Socket: A Prospective Study

**DOI:** 10.5681/joddd.2010.027

**Published:** 2010-12-21

**Authors:** Reza Khorshidi Khiavi, Maghsood Pourallahverdi, Ayda Pourallahverdi, Saadat Ghorani Khiavi, Sina Ghertasi Oskouei, Hadi Mokhtari

**Affiliations:** ^1^ Member of Scientific Board, Department of Oral & Maxillofacial Surgery, Faculty of Dentistry, Tabriz University of Medical Sciences, Tabriz, Iran; ^2^ Anesthesiologist, Department of Anesthesiology, Sina Hospital, Tabriz University of Medical Sciences, Tabriz, Iran; ^3^ Private Practice, Tabriz, Iran; ^4^ Faculty of Dentistry, Tabriz University of Medical Sciences, Tabriz, Iran; ^5^ Assistant Professor, Department of Endodontics, Faculty of Dentistry, Tabriz University of Medical Sciences, Tabriz, Iran

**Keywords:** Bupivacaine, irrigation, pain, third molar

## Abstract

**Background and aims:**

The surgical removal of the lower third molars is a procedure generally followed by side effects such as postoperative pain. The aim of this study was to evaluate the efficacy of socket irrigation with an anesthetic solution in relieving pain following impacted third molar surgery.

**Materials and methods:**

Thirty-four patients (17 males and 17 females), aged 18-24 years, with bilateral impacted lower third molars were selected. Both third molars were extracted in one surgical session. Tooth sockets in each patient were rinsed randomly either with 4 mL of 0.5% bupivacaine hydrochloride plain (without vasoconstrictor) anesthetic solu-tion or 4 mL of normal saline, used as control. The patients were instructed not to use analgesics as long as possible, and if not, they were instructed to use an analgesic, and record the time. Pain severity was assessed using a visual analogue pain scale (VAPS) at 1-, 6-, 12-, and 24-hour intervals post-operatively. Data were analyzed using Pearson’s chi-square test and P <0.05 was considered statistically significant.

**Results:**

Post-operative pain difference between the two groups was statistically significant at 1-, 6-, 12- and 24-hour post-operative intervals (P <0.05). Post-operative pain increased in both groups to a maximum 12 hours after surgery with signif-icant improvements after that.

**Conclusion:**

Based on the results, the irrigation of surgery site with bupivacaine after third molar surgery significantly reduces post-operative pain.

## Introduction

Third molars generally erupt between 18 and 24 years of age, with wide variations in the eruption time, and the eruption failure is very common, which makes the extraction of impacted third molars one of the most frequent surgical procedures carried out in the world.^1,2^ The surgical removal of the lower third molars is a procedure generally followed by side effects such as postoperative pain, swelling, and trismus.^3-7^ Post-operative pain is the most common complication after tooth extraction.^8-10^ According to literature, pain after surgical extraction of a third molar reaches its highest intensity 6-8 hours after surgery.^11,12^

There are several methods for post-operative pain relief.^13,14^ Recently, a novel approach has been introduced for the delivery of a local anesthetic agent into the tooth socket instead of the use of a blocker or analgesic agent such as non-steroidal anti-inflammatory drugs (NSAIDs). The technique consists of socket irrigation with the anesthetic agent after tooth extraction.^15^ Potential complications are minimal since neither a needle nor an injection is involved. Consequently, the risk of intravascular or intra-neural injection of the anesthetic agent is extremely remote;^16, 17^ in addition, there is no need for administration of drugs such as NSAIDs, which results in complications like gastrointestinal lesions.^18^

We postulated that a similar technique might be effective in relieving post-operative pain after surgical tooth extraction. To test this hypothesis, we designed a prospective, randomized, double-blind clinical trial to study the effect of socket irrigation with 0.5% bupivacaine hydrochloride plain solution on reducing the severity of post-operative pain after surgical tooth extraction.

## Materials and Methods

Consecutive patients undergoing surgery for third molar extraction in the Department of Oral and Maxillofacial Surgery, Faculty of Dentistry, Tabriz University of Medical Sciences, Tabriz, Iran, were enrolled in this study. Inclusion criteria were:

- Age between 18 and 24 years
- Bilateral impacted lower third molars (class B and 2 of Pell & Gregory classification^19^)
- No systemic disease
- No history of allergic reactions to local anesthetic agents

Individuals who used analgesics were not included, since variability in the effects of drugs on subsequent pain levels could not be assessed accurately. Equal numbers of males and females were enrolled in the study. The study procedure as well as the probable risks and discomforts were explained to the patients. They were informed that they could withdraw from the study any time they desired. A written informed consent was obtained from each participant before the study. The study design was approved by the Research and Ethics Committees of Tabriz University of Medical Sciences.

No pre-medications were used. All the surgeries were carried out by one experienced oral and maxillofacial surgeon. In all of the patients, both impacted lower third molars were surgically extracted in one session. Both sides were randomly assigned to either the study or the control group. The patients’ blood pressures were registered 30 min before the surgery. Assignment to the study group meant that a disposable syringe containing 4 mL of 0.5% bupivacaine hydrochloride plain (Merck, Darmstadt, Germany), which does not contain vasoconstrictors, was prepared. In the control group, 4 mL of normal saline was placed in the syringe. Neither the operating surgeon nor the assistant was aware of the contents of the syringe. The nursing personnel were made aware that a study was in progress; however, they were not told which irrigation solution each surgery site received. The contents of the syringes and the amount of solution used were recorded in the patients’ file. Before closure of the wound at the end of the surgery, the socket was rinsed with either one of the solutions. After irrigation, the wound was sutured. Patients were asked to stay in the waiting room for 30 min after surgery, when their blood pressures were measured again. The patients were recommended not to use analgesics as long as possible. If needed, the patients could use acetaminophen and record the time of its use, in order to be excluded from the study after this time.

Pain severity was assessed using a visual analogue pain scale (VAPS) at 1-, 6-, 12-, and 24-hour intervals post-operatively by the assistant surgeon, who was still unaware of irrigation solutions each side had received. The assistant questioned the patients with respect to nausea and their pain severity on each side. Patients were instructed to say a number (0-10) for their pain intensity on each side. The patients were aware that the scale served to analyze the presence and intensity of pain alone and was not a representation of generalized post-operative discomfort. Post-operative pain was assessed in a double-blinded manner; neither the patient nor the assistant was aware of the solution administered to each side.

Data were assessed using Pearson’s chi-square test. In this study, p<0.05 was considered statistically significant.

## Results

A total of 34 patients, 17 females and 17 males (mean age 20.62 ± 1.67 years, range 18–24 years), participated in this study. Surgical operations were carried out on a total of 68 third molars (34 right and 34 left lower third molars).

**Figure 1:**
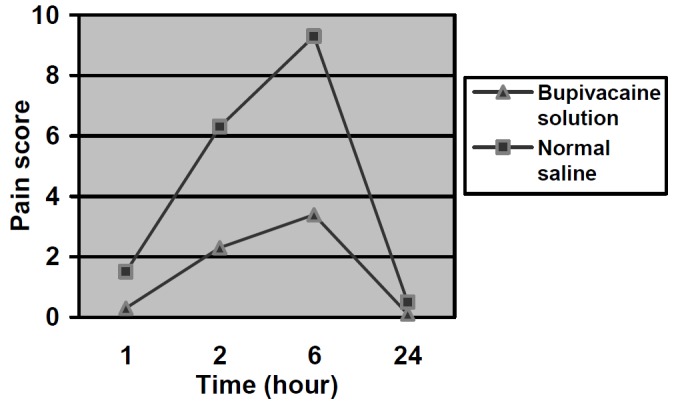
Pain level recorded at four time intervals post-operatively in the test and control groups.

Bupivacaine irrigation was administered on 17 right and 17 left tooth sockets after surgical extraction. Two patients were excluded from the study because they used an analgesic. Post-operative pain, as recorded on VAPS, was significantly less in the group receiving bupivacaine irrigation compared with the controls (Table 1). The difference was statistically significant at 1-, 6-, 12- and 24-hour intervals post-operatively (p<0.05). Post-operative pain increased in both groups to a maximum 12 hours post-operatively, with significant improvements after this time (Figure 1). Apart from post-operative pain, there were no significant complications in either the case or control groups. Specifically, there were no signs of bupivacaine toxicity such as cardiovascular alterations leading to changes in blood pressure or vomiting in any patient.

**Table 1:** Means and standard deviations (mean ± SD) of pain severity according to scores of visual analogue scale in the test and control groups

Rinsing solution	Pain recorded post-operatively			
	1 hour	6 hour	12 hour	24 hour
Bupivacaine	0.3 ± 0.87	2.59 ± 0.97	3.89 ± 0.93	0.1 1± 0.32
Normal saline	1 ± 1.07	5.78 ± 1.34	8.00 ± 1.71	0.48 ± 0.64

## Discussion

The present study evaluated the efficacy of socket irrigation with bupivacaine in comparison with normal saline irrigation in reducing post-operative pain severity associated with third molar surgery. Moderate-to-severe pain often accompanies third molar surgery during the first 12 hours after surgery.^20-22^ In many cases, this requires oral narcotic analgesics for pain relief. An alternative method to the oral administration of medications for achieving analgesia has been well known since late 19th century: local injection of an anesthetic agent. However, local injection of anesthetic agents is associated with complications such as nerve injury, vascular injury, spread of infection, and intravascular or intra-neural injection of the anesthetic agent.^16, 17^

A method for delivery of local anesthetics during surgery has recently been described, which involves irrigation of the socket with an anesthetic solution using a blunt-tipped catheter after tooth extraction. This method successfully delivers the anesthetic agent. Because no injection or needle is involved, the potential risks of the procedure are limited to hypersensitivity reaction to the anesthetic agent.^15^

Bupivacaine hydrochloride is an anilide anesthetic agent similar to lidocaine but has a much longer duration of action. Since its introduction in the United States in the 1970s, it has been tested extensively. Bupivacaine’s duration of action of up to 12 hours makes it an ideal medication for use in surgical procedures, such as impacted tooth extraction, in which the bulk of the post-operative discomfort occurs within the first 24 hours.^16, 20, 21^ Some complications of bupivacaine are cardiac toxicity, nausea, paroxysm, and vertigo, but these complications are related to plasma concentrations of bupivacaine.^23, 24^

Our study indicates that socket irrigation with 0.5% bupivacaine hydrochloride plain provides effective pain relief in the majority of patients undergoing third molar surgery. In addition, the use of such socket irrigation appears to be safe in individuals without a history of hypersensitivity to local anesthetic agents or cardiac complications. This method is also simple, convenient, and cost-effective.

Several studies have investigated the effects of local anesthetic agents used to irrigate or to be injected into the surgery zone for post-operative pain relief.^25,26^ One study investigated the efficacy of local anesthetic infusion for pain control after cesarean section and found that there were no significant differences in patient demographics or visual analog pain scales at any time interval between the bupivacaine and placebo groups. However, narcotic requirements to produce this amount of pain relief were significantly less in patients who received bupivacaine infusion rather than normal saline solution at all time intervals.^26^ In another study comparing the effect of mandibular nerve block with bupivacaine versus lidocaine for pain relief after third molar surgery, it was reported that bupivacaine significantly reduced post-operative pain experience only at an 8-hour period. No difference in analgesic requirements or cardiovascular responses was observed with the respective toxic threshold concentrations. Since an injection method was employed, factors like infection and distance from the site of injection could affect the results. Moreover, the intensity of pain in the bupivacaine and lidocaine groups was assessed in different subjects, which introduces the effect of individual differences in pain perception on interpretation of the results. Therefore, the findings could not be as reliable as it was performed in the present study, i.e. assessments on two sides of the mouth in the same patient.^27^ In another study investigating the effect of intra-operative bupivacaine irrigation for management of shoulder-tip pain following laparoscopy, it was shown that mean shoulder-tip pain scores as recorded on visual analogue pain scale were significantly lower in the bupivacaine group than in the control group 4 to 24 hours after surgery. Post-operative analgesia requirement also significantly decreased in patients receiving bupivacaine irrigation.^28^ In addition, the efficacy of retrobulbar bupivacaine irrigation for relieving post-operative pain after scleral buckling surgery was evaluated in a study which revealed this method is a safe and effective way to achieve post-operative pain relief after surgery for scleral buckling.^16^ In the two previously mentioned studies, patients were under general analgesia, which may bias the results. The design of the study also involved evaluation of different individuals which involves the afore-mentioned limitation.

In our study, third molar surgery on two sides was performed in one patient simultaneously by one surgeon. One surgery site was rinsed with bupivacaine solution and the other with normal saline. Such design in the study allowed unbiased comparison of post-operative pain intensity with regard to differences in pain perception between individuals.^29-31^ In addition, patients who took analgesics were excluded from the study, which eliminated the interventional effect of medications such as NSAIDs from the results.

According to the findings of the present study, it can be concluded that socket irrigation with bupivacaine significantly reduces post-operative pain following third molar surgery. In conclusion, the results of this study demonstrate the effectiveness of bupivacaine irrigation in reducing post-operative pain 1, 6, 12, and 24 hours after surgery without any significant complications.
